# Slug Monitoring and Impacts on the Ground Beetle Community in the Frame of Sustainable Pest Control in Conventional and Conservation Agroecosystems

**DOI:** 10.3390/insects11060380

**Published:** 2020-06-18

**Authors:** Davide Scaccini, Michela Panini, Olga Chiesa, Rinaldo Nicoli Aldini, Vincenzo Tabaglio, Emanuele Mazzoni

**Affiliations:** 1 Department of Sustainable Crop Production, Università Cattolica del Sacro Cuore, Via Emilia Parmense 84, I-29122 Piacenza, Italy; michela.panini@unicatt.it (M.P.); olga.chiesa@unicatt.it (O.C.); rinaldo.nicoli@unicatt.it (R.N.A.); vincenzo.tabaglio@unicatt.it (V.T.); 2 Department of Agronomy, Food, Natural Resources, Animals and Environment, University of Padova, Viale dell’Università 16, 35020 Legnaro, Italy

**Keywords:** *Deroceras invadens*, cover crop, conservation agriculture, monitoring, beer traps, *Pterostichus melas*, *Poecilus cupreus*

## Abstract

In conservation agriculture, slugs are considered significant pests and their monitoring is a key option in the integrated pest management framework. Together with molluscicide applications, predators such as ground beetles can offer a tool for slug control in the field. Through the evaluation of slug and ground beetle monitoring strategies, this work compared their presence in conventional and conservation agricultural plots. The invasive *Deroceras invadens* was the dominant slug species to occur in all sampling periods. Among Carabidae, *Poecilus cupreus* and *Pterostichus melas* were the most abundant species, and *Bembidion* spp., *Brachinus* spp., and *Harpalus* spp. were also common. Beer-baited pitfall traps, whatever their alcoholic content, caught more slugs and ground beetles than wooden boards used as shelters. Slugs were more abundant in conventional plots than in conservation plots, possibly due to the lower presence of natural enemies such as ground beetles. Despite possible impacts on Carabidae, beer-baited pitfall traps should be considered a useful tool for slug monitoring and for the planning of molluscicide applications. Soil management such as minimum- or no-tillage and the presence of cover crops are important elements influencing both slug and ground beetle presence, possibly playing a key role in the maintenance of natural enemy populations.

## 1. Introduction

In agroecosystems, negative effects due to landscape simplification could be mitigated by adopting conservation agricultural practices, such as no-tillage, cover cropping, crop rotation, residue management [1,2,3], and promoting ecosystem services such as biological control [4,5]. In sustainable agriculture, the reduction of soil tillage, as well as increasing the presence of crop residues, may create favourable conditions for some pests, increasing their occurrence and / or influencing their population dynamics [6,7,8,9,10]. This can also be true for pestiferous slugs [11,12,13]. Many organisms are more abundant in no-tillage than in conventional tillage systems, where larger organisms are in general more sensitive to soil tillage disturbance as compared to smaller ones [14]. Soil tillage is known to affect soil functions, and a high intensity of tillage often allows for better pest control [15], also influencing differences in weed presence and dynamics. Indeed, practices based on non-inversion tillage have been shown to increase weed infestation and to modify the biological community of the soil [16].

In conservation agricultural systems, slugs can use weeds as an alternative food source, as is also true for to the plants seeded for intercropping [17,18,19,20]. In addition, the presence of crop residues on the soil surface may provide a suitable habitat for these pests [13,21], increasing their feeding activity [22]. In fact, slug damage, which is most severe in periods when plants are more susceptible (i.e., seedlings, after emergence), increases in cool, wet conditions [13] as is the case when residues are present on the soil surface [23]. Nevertheless, in no-till cultivation slug presence and damage tends to decrease following an appropriate transition period [24,25], when a new equilibrium between biological communities in no-till-soil is reached. Cover crops and reduced soil tillage can increase the abundance of slugs’ natural enemies such as ground beetles (Coleoptera: Carabidae) [26,27], which are generally more abundant under this type of management than in ploughed fields [28,29,30,31]. In many agricultural environments, some carabid species act as predators of slugs, thus providing an ecosystem service as biological control agents against these pests [26,27].

Together with the preservation of natural enemies that may prove key in slug control in crop production, monitoring practices are crucial in an integrated pest management (IPM) framework [32], especially before and after crop seeding [33,34,35]. Knowledge of the infestation levels and biology of slugs is vital in order to achieve effective IPM strategies in agroecosystems, also providing viable management solutions for farmers [13]. In IPM, sampling can provide real-time quantification of pest abundance and, when referring to a threshold, it can modulate the decision making for management options [32,36,37].

The aims of this study were: (i) to verify the efficacy of different monitoring tools (i.e., wooden boards and two beer traps with different alcohol contents) for monitoring slugs, and assess their impact on the ground beetle community, and (ii) to study slug and ground beetle presence in a long-term experimental trial focused on a comparison between conventional and conservation agriculture.

## 2. Materials and Methods

### 2.1. Trial No. 1: Efficacy of Traps and Refuges in Monitoring Slugs and Ground Beetles in Conventional and Conservation Agroecosystems

Starting from 2011, a field experiment comparing conventional agriculture (treatment CONV, based on ploughing and without cover crops) and conservation agriculture (treatment CONS, based on no-tillage and cover cropping) was carried out at the CERZOO Experimental Farm (45°00′22′’ N, 9°42′24′’ E, 70 m a.s.l.) in San Bonico, Piacenza (northern Italy). A three-year crop rotation (maize, soybean, winter wheat) was adopted for both treatments. According to the rotation scheme, in the conservation plots, three different cover crops were sown: rye (*Secale cereale* L.), hairy vetch (*Vicia villosa* Roth.) and a mixture of rye, hairy vetch, Italian ryegrass (*Lolium multiflorum* var. *italicum* Lam.), crimson clover (*Trifolium incarnatum* L.), phacelia (*Phacelia tanacetifolia* Benth.), and radish (*Raphanus raphanistrum* subsp. *sativus* (L.) Domin). The trial was arranged in a completely randomized block layout with four replications and four treatments. The treatments were: (i) CONV, conventional tillage without cover crop; (ii) CONS-Rye, no-tillage with cover crop of rye; (iii) CONS-Vetch, no-tillage with cover crop of hairy vetch; (iv) CONS-Mix, no-tillage with cover crop of mixture. Each plot was 22 m wide and 65 m long.

A survey of the field to monitor slug presence was carried out from 26 March 2015 to 1 June 2016. The following refuges and traps to monitor slug presence were evaluated: (i) wooden board (30 × 30 cm); (ii) beer trap A with 5.7% alcohol; (iii) beer trap B with 8.6% alcohol. Beer traps were set in common pitfall traps (200 mL of volume), partially covered by plastic dishes. Traps / refuges were placed in the midline of the plots, one type for each replicate, and located at least 15 m from each other. Traps were exposed in the field for a week, and then removed for inspection and maintenance. A first sampling round was performed in spring 2015, from 26 March to 25 May (5 samplings); a second sampling round (2 samplings) was carried out in October 2015; the last round was performed in spring 2016, from 3 March till 1 June (6 samplings). In other sampling events, data collection was compromised due to trap damage (see below).

### 2.2. Trial No. 2: Efficacy of Traps and Refuges in Monitoring Slugs and Ground Beetles in a No-Till Field

A second trial was performed in a 3 ha field converted the previous year to no-tillage just after the setting up of an SDI (subsurface drip irrigation) system. This field was also located at the CERZOO Experimental Farm. Surveys on this field started on 19 November 2015 and ended on 1 June 2016 (8 valid samplings were available). Two strips 20 m wide were marked out in the field. Wooden boards and beer traps as previously described (Section 2.1) were placed at least 15 m from each other, along the middle of each strip, randomised and replicated four times.

### 2.3. Data Collection and Species Identification

The slugs under the wooden board refuges and inside the beer traps were collected and counted weekly. The specimens were morphologically identified to their species using identification keys evaluating external features and genitalia of dissected specimens [38,39,40]. The identification of two species was confirmed on some randomly selected specimens by mtDNA analysis (see below).

The ground beetles in the beer traps and the under wooden boards were counted and identified at least to the genus level, or to the specific level for the most common species with a predatory behaviour towards slugs [41].

During samplings, on some occasions, beer traps were found to have been damaged by wild fauna, in particular wild boars (*Sus scrofa* L.), red foxes (*Vulpes vulpes* (L.)) and roe deers (*Capreolus capreolus* (L.)). Their activity against these traps was ascertained by evidence in the form of tracks, faeces inside traps (for foxes), or by direct observation, above all for roe deer. For this reason, statistical analyses were performed on datasets where all trap combinations were available, and excluding dates with missing values.

### 2.4. DNA Barcoding

Live slug specimens collected in the field were killed by freezing and then preserved in ethanol, acetone or kept frozen at −20 °C. For DNA analysis, a portion of slug foot (~2 mm^3^) from some randomly selected specimens was cut away, avoiding the inclusion of material from the digestive tract [42,43]. DNA extraction was performed using a “salting out” protocol [44]. Extracted DNA was used to amplify mitochondrial cytochrome c oxidase subunit I (COI) gene, following a published protocol [45]. Primers LCO1490 (5′-GGTCAACAAATCATAAAGATATTGG-3′) and HCO2198 (5′-TAAACTTCAGGGTGACCAAAAAATCA-3′) were used [46]. Amplicons were purified using GenElute™ PCR Clean-Up Kit (Sigma-Aldrich, Milan, Italy), sequenced in both strands, and compared with GenBank database using BLAST.

### 2.5. Statistical Analysis

Statistical analysis on slug and ground beetle captures was performed using the IBM SPSS Statistics package release 25. A general linear model repeated measures procedure was used considering sampling dates as the “within factor” variable and trap and (when applicable) soil management as “between factor” variables. A Greenhouse–Geisser correction of degrees of freedom was adopted when the assumption of sphericity was not met (Mauchly’s Test). Means of “between factors” were separated using the Student–Newman–Keuls *post hoc* test (SNK). An alpha level of 0.05 was used for all statistical tests. Results of the statistical analysis are available in Supplementary Table S1 (trial no. 1) and S2 (trial no. 2).

## 3. Results

### 3.1. Trial No. 1

In the first trial, 1702 slugs were captured. They belonged to the following families: Agriolimacidae (2 species), Arionidae (1 species) and Limacidae (2 species). The most abundant slug was the invasive *Deroceras invadens* Reise, Hutchinson, Schunack and Schlitt (Agriolimacidae) (*n* = 1665; 97.8%), followed at some distance by *Limax maximus* L. (Limacidae) (*n* = 24; 1.4%) and *Deroceras reticulatum* (Müller) (Agriolimacidae) (*n* = 10; 0.6%). Just a few specimens of *Ambigolimax valentianus* (Férrussac) (Limacidae) (*n* = 2; 0.1%) and *Arion* sp. (Arionidae) (*n* = 1; < 0.1%) were found.

**Figure 1:**
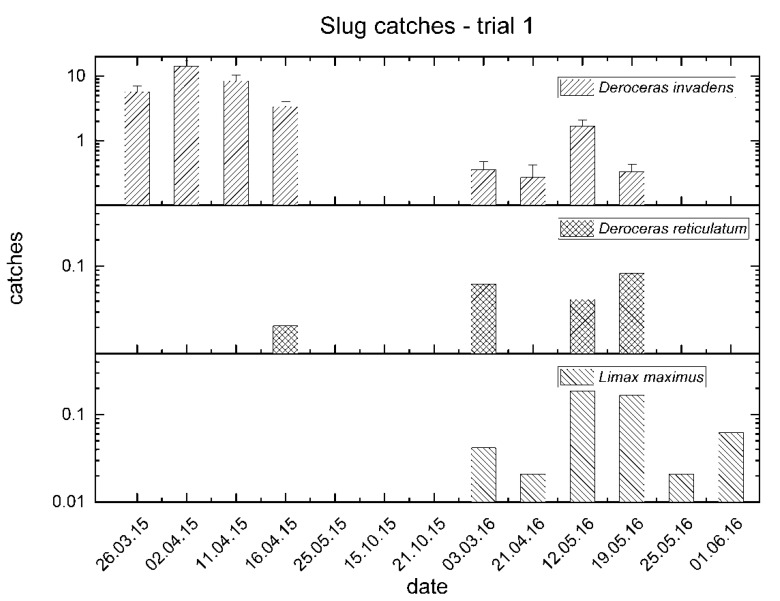
Mean catches of slugs between March 2015 and June 2016 in trial no. 1.

Captures of *D. invadens* were the highest in March–April 2015 (slightly less than 15 specimens per trap), when in some cases a few individuals were observed in courtship behaviour under wooden boards. Only one specimen was captured in October 2015, while more specimens were collected in the period March–June 2016. An increase in catches was observed in May 2016 (Figure 1). *L. maximus* was mainly recorded in the period March–May 2016, and a similar trend was observed for *D. reticulatum* (Figure 1).

The analysis of variance (repeated measure ANOVA) was performed on capture data for *D. invadens*. The statistical analysis was not performed on catches of the other species due to the low level of captures.

Catches of *D. invadens* were significantly affected by the sampling date (F_1.80,64.93_ = 32.92; *p* < 0.001). Interactions of sampling date with the type of trap (F_3.61,64.93_ = 7.92; *p* < 0.001) and soil management (F_5.41,64.93_ = 8.12; *p* < 0.001) were also significant, while the full interaction “date / trap / management” was slightly significant (F_10.82,64.93_ = 2.35; *p* < 0.05).

A significantly higher number of *D. invadens* individuals were found in the conventional plots (CONV) (F_3,36_ = 7.29; *p* < 0.01). Among conservation treatments (CONS), more individuals were found in rye plots than in those with vetch or mixture, though the difference was not significant (Table 1). No significant differences were detected between the two types of beer trap (Table 2), which collected significantly more slugs than wooden refuges (F_2,36_ = 9.55; *p* < 0.01). No significant interaction between trap type and soil management was found (F_6,36_ = 2.36; NS).

The ground beetles collected in the traps or under the wooden boards reached very high numbers: 17,900 specimens in total. They were grouped according to their feeding behaviour as follows: predatory species such as *Pterostichus* Bonelli [28,47,48,49], generalist predators / polyphagous such as *Poecilus* Bonelli [49,50,51], and omnivorous / granivorous species such as those belonging to the genera *Bembidion* Latreille, *Brachinus* Weber and *Harpalus* Latreille [52,53,54,55,56]. The most common species was *Poecilus cupreus* (L.) (*n* = 12,161; 67.9%). *Pterostichus (Feronidius) melas* (Creutzer) catches totalled 1573 (8.8%). The remaining specimens (*n* = 4166; 23.3%) belonged to the genera *Bembidion*, *Brachinus*, *Calosoma* Weber and *Harpalus*, the latter with *Harpalus distinguendus* (Duftschmid) (*n* = 116; 0.7%) and *H. (Pseudoophonus) rufipes* (De Geer) (*n* = 30; 0.2%). The largest species found was *Calosoma (Campalita) maderae* (Fabricius), but only four specimens were collected.

**Figure 2:**
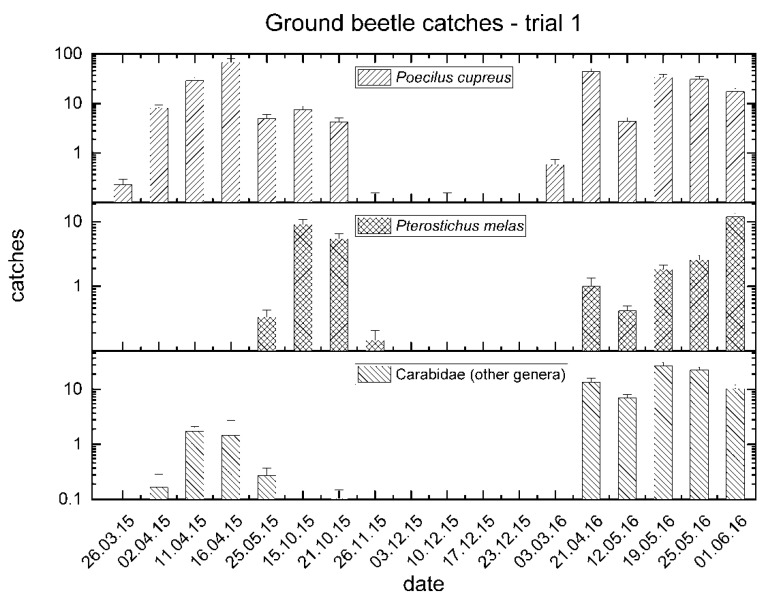
Mean catches of ground beetles between March 2015 and June 2016 in trial no. 1.

The captures of *P. cupreus* were higher in spring and autumn, with similar patterns in 2015 and 2016. Catches of *P. melas* and of other genera showed a similar trend with slight differences, such as reduced captures of *P. melas* in spring 2015 and no catches of other genera in autumn 2015 (Figure 2).

Catches of *P. cupreus*, *P. melas,* and of all the other genera, grouped together, were analysed with repeated measure ANOVA to evaluate the effect of sampling date, trap type and soil management.

Catches of *P. cupreus* were significantly affected by the sampling date (F_3.47,124.85_ = 47.59; *p* < 0.001). Also, the interactions of sampling date with type of trap (F_6.94,124.85_ = 12.22; *p* < 0.001) and with soil management (F_10.40,124.85_ = 6.99; *p* < 0.001), as well as the full interaction “date / trap / management” (F_20.81,124.85_ = 2.77; *p* < 0.001) were significant. *P. cupreus* catches were also significantly affected by the “between subject effects”: type of trap (F_2,36_ = 87.66; *p* < 0.001) and soil management (F_3,36_ = 9.02; *p* < 0.001), but only marginally by the interaction between these factors (F_6,36_ = 2.97; *p* < 0.05).

Catches of *P. melas* were significantly affected by the sampling date (F_2.77,99.53_ = 42.96; *p* < 0.001). A significant interaction of sampling date with type of trap was observed (F_5.53,99.53_ = 11.93; *p* < 0.001) but not with soil management (F_8.92,99.53_ = 1.56; NS) nor with the full interaction “date / trap / management” (F_16.59,99.53_ = 1.12; NS). Considering the “between factors” of the model, the type of trap significantly influenced catches of *P. melas* (F_3,36_ = 47.03; *p* < 0.001) (Table 2) but soil management was only slightly significant (F_3,36_ = 2.93; *p* < 0.05) (Table 1) and the interaction of the two factors was not significant (F_6,36_ = 1.98; NS).

The analysis of catches for the other groups of Carabidae revealed that they were significantly affected by the date (F_2.98,107.41_ = 45.89; *p* < 0.001). As in previous cases, a significant interaction of sampling date with type of trap was detected (F_5.97,107.41_ = 12.06; *p* < 0.001), but not with soil management (F_8.95,107.41_ = 1.46; NS) nor with the full interaction “date / trap / management” (F_17.90,466.28_ = 1.32; NS). A significant trap effect was present (F_2,36_ = 50.34; *p* < 0.001). Little influence due to soil management was observed (F_3,36_ = 4.20; *p* < 0.05) and, as in the previous cases, factor interaction was not significant (F_6,36_ = 1.42; NS).

Differences between treatments were observed for *P. cupreus* and for the Carabidae aggregation of different species: a significantly lower level of catches was observed in CONV plots and the highest levels were obtained in CONS-Vetch plots. Catches of *P. melas* were more uniformly distributed and without significant differences due to soil management practices and cover crops (Table 1). Similar to slugs, catches of *P. cupreus* were higher in beer traps than under wooden boards. The same situation was observed with other Carabidae (Table 2).

### 3.2. Trial No. 2

A lower number of slugs were collected during the survey in the SDI field. In total, 281 slugs were captured, belonging to the families Agriolimacidae (2 species), Limacidae (2 species), Milacidae (1 species) and Arionidae (1 species). They were mostly represented by *D. invadens* (*n* = 241; 85.8%) and, to a less extent, *Tandonia budapestensis* (Hazay) (Milacidae) (*n* = 28; 10.0%) and *A. valentianus* (*n* = 8; 2.8%). Only occasionally, *D. reticulatum* (*n* = 1; 0.4%), *L. maximus* (*n* = 2; 0.7%), and *Arion* sp. (*n* = 1; 0.4%) were captured.

**Figure 3:**
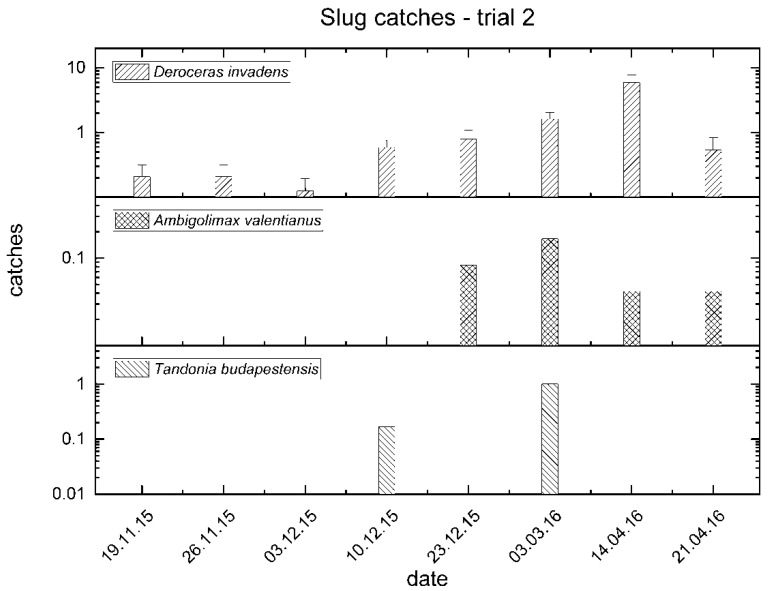
Mean catches of slugs between November 2015 and April 2016 in trial no. 2.

A continuous presence of *D. invadens* was observed in the period from November 2015 to April 2016, with a peak of presences in mid-April (Figure 3). Only a few individuals of *A. valentianus* were captured from December to April (Figure 3). *T. budapestensis* was caught in high numbers between the end of February and the beginning of March, coinciding with significant catches of the previous species, and some specimens were also found in December (Figure 3).

The analysis of variance (repeated measure ANOVA) performed on data for *D. invadens* revealed a significant difference in catches between sampling dates (F_1.232,25.872_ = 8.51; *p* < 0.01) but no significant interactions between sampling date/type of trap were found (F_2.464,25.872_ = 1.99; NS). No significant differences were detected between the catches with the three types of trap (F_2,21_ = 1.37; NS) (Table 3).

**Figure 4:**
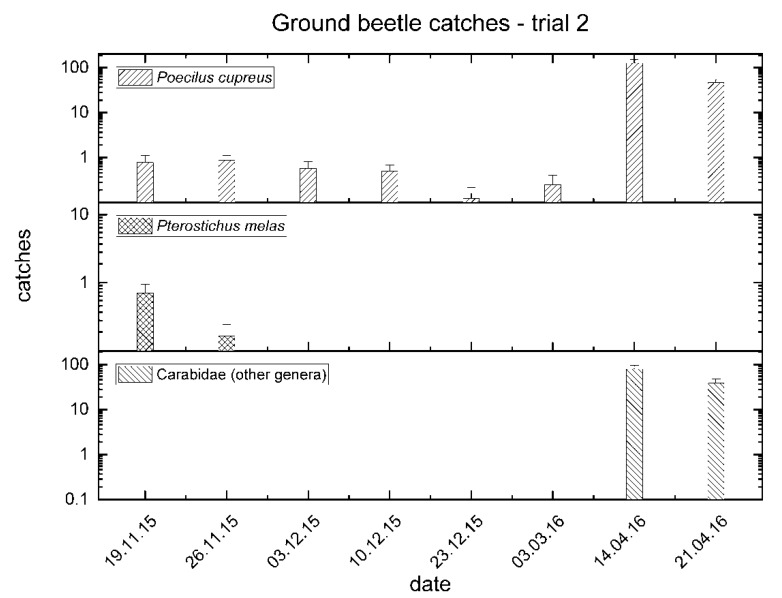
Mean catches of ground beetles between November 2015 and June 2016 in trial no. 2.

The total number of Carabidae caught was 7065. Of these, *P. cupreus* was the most abundant in April 2016, with more than 100 catches per trap, although this species was also present during winter. Overall, 4206 *P. cupreus* were captured, corresponding to 59.5% of the ground beetle fauna collected in the SDI field. *P. melas* catches were much lower (only 24; 0.3%), and occurred mainly in autumn and not in spring. On the contrary, specimens of other genera of carabids were collected only in spring (*n* = 2835; 40.1%) (Figure 4).

During this trial, Carabidae were captured only in beer traps. The sampling date always affected the number of captured specimens of *P. cupreus* (F_1.13,23.69_ = 54.48; *p* < 0.001), with a significant interaction with the type of trap (F_2.26,23.69_ = 14.14; *p* < 0.001). Significant differences were detected between the catches with the three types of trap (F_2,21_ = 38.81; *p* < 0.001) (Table 3).

*P. melas* catches were significantly different when considering the sampling date (F_1.37,28.74_ = 7.47; *p* < 0.01), but the interaction between sampling / trap type was not significant (F_2.74,28.74_ = 2.53; NS). Catches of this species were slightly affected by the trap model (F_2,21_ = 3.50; *p* < 0.05) (Table 3).

The captures of other genera of Carabidae were statistically different on different dates (F_1.68,35.32_ = 44.35; *p* < 0.001), with a significant interaction with trap model (F_3.36,35.32_ = 11.65; *p* < 0.001). Furthermore, a significant effect of the trap model on catches was confirmed (F_2,21_ = 28.68; *p* < 0.001) (Table 3).

### 3.3. Slug DNA Barcoding

Four specimens of *D. invadens* and two of *A. valentianus* were analysed, and the COI sequences confirmed the morphological identification of both species.

## 4. Discussion

*D. invadens* was the most common species found in both field trials. Damage to maize seedlings occurred two years before the start of this study, when the crop was compromised in only 2–3 days [57], presumably related to the feeding activity of this slug. Observations made during the monitoring period confirmed its pest status on the investigated crops. *D. invadens* is an invasive pest species in the field, able to adapt to areas disturbed by human activity such as gardens or close to discarded rubbish and highly polluted rural areas [58,59]. Our observations found that this species was also active during winter, probably related to its ability to adapt to cool temperatures and frequent rainfall (Supplementary Figure S1). On the other hand, sampling carried out during summer did not record any evidence of this slug. Other slug species found during the field activity are common pests, often introduced into new areas by human activities, as in the case of *L. maximus* and *T. budapestensis* [40,60]. Despite the well-known pest status of *D. reticulatum* on crops and ornamental plants, findings of this species were just occasional here. Furthermore, we found only a few specimens of *A. valentianus*, a common pest species in fields, gardens and greenhouses [40], whose control should be evaluated in the event of high infestation.

DNA barcoding was used to support specific identification of *D. invadens*, which mainly requires the analysis of genitalia by dissection to distinguish it from *Deroceras laeve* (Müller) [38,43,61]. Analysis of genitalia is also necessary for *A. valentianus*, to distinguish this species from *Ambigolimax nyctelius* (Bourguignat) [43,62]. In all cases, barcoding confirmed the morphological identification.

As far as ground beetles are concerned, *P. cupreus* and *P. melas* were the dominant carabid species in the investigated fields. Previous studies on the ground beetle community in the area showed comparable data in species abundance, although with a higher presence of *P. melas* (38.4%) than *P. cupreus* (22.0%) [63]. Here, we found *P. cupreus* individuals mainly in spring, with few specimens in autumn and winter. *P. cupreus* is indeed a spring breeding species [64] that also occurs in summer and autumn [65,66], when numbers may change according to the different field management systems adopted [30,31,67]. Individuals of *P. melas*, an autumn breeder in agricultural areas [68], were observed mainly in spring and autumn (only in autumn for trial no. 2), while the other carabid species occurred almost exclusively in spring. The presence of Carabidae during periods of slug activity and, in particular, reproduction may enhance predation against these pests, thus helping with their IPM role, playing as natural enemies. It should be noted that, even if no direct evidence of slug predation by Carabidae was observed during monitoring, ground beetles often prefer to feed on slug eggs and juveniles if compared with large, adult slugs [48,69,70,71].

Beer-baited pitfall traps captured more slugs than did refuges. It is worthwhile specifying that catches are additive for beer traps during the exposure time (seven days in this study), while refuges normally host slugs for some periods of the day; this could partially explain the lower number of specimens found after the same exposure time. Other refuges used during a preliminary set-up of this study, such as terracotta roof tiles, proved to not be suitable for slug monitoring, probably due to the increased temperature below them after exposure to sunlight. In slug monitoring and control, beer-baited pitfall traps follow the indications given by Young et al. [72] to prefer non-toxic baits to molluscicide baits. The attractiveness of beer for slugs, as reported long ago by Taylor [73] for *D. reticulatum* and other pest species, is probably due to the presence of diacetyl, acetoin, dihydroxyacetone [74], and decanoic acid [75]. No differences between the two beers used in this study in terms of slug catches were recorded. Indeed, differences in alcohol content probably have no effect as regards slug captures [76]. Though beer-baited traps are commonly used in gardens, vegetable gardens, and fields [13], their use in open field conditions should be carefully evaluated, because they need frequent monitoring (i.e., refilling / changing the beer, removing dead specimens), and can be damaged by wild fauna. Indeed, the damage observed on pitfall traps led to a failure in data collection, in particular due to fox, wild boar and roe deer activity. Roe deer and wild boars are also known to cause crop damage in field conditions [77]. Based on these observations, some precautions in setting up beer-baited pitfall traps are suggested, such as adding weights and iron cages, thus permitting insect and slug samplings and avoiding or significantly reducing damage by wild fauna.

As with the slug catches, in both trials the number of Carabidae found in beer traps was consistently higher than for wooden refuges. Almost 18,000 ground beetles were collected in trial no. 1, and more than 7000 in trial no. 2. In fact, pitfall traps with or without baits are widely used for Carabidae collecting [78,79] and the use of beer as a bait is a well-known practice in entomological studies, beer being very attractive for ground beetles [80]. However, the use of beer-baited pitfall traps for slug collection in agricultural fields should be discouraged for long term monitoring, because of its potential impact on the ground beetle fauna, as evidenced here.

It is noteworthy that some differences in slug presence were recorded between soil management systems; specifically, more *D. invadens* individuals were found in conventional plots than in conservation plots (with the exception of plots with rye as cover crop, which showed intermediate levels of catches). Similarly, in all conservation treatments *L. maximus* was generally less frequent than in conventional plots. These results seem surprising due to the known positive relationship between slug abundance and fields managed with minimum / no-tillage and presence of mulch / cover crops [13,23,29]. However, in conservation agricultural systems slugs are often less abundant than in systems involving high-disturbance tillage, partly due to an increase in natural enemies that act as biological control agents, mitigating their damage [26,27,81]. Moreover, the fields surveyed underwent an appropriate transition period from conventional management, leading to a new equilibrium between biological communities that may decrease slug presence [24,25]. Indeed, natural enemies such as Carabidae and other predators (as Coleoptera: Staphylinidae and Opiliones) were often found in the conservation fields we surveyed. Carabidae are generally less abundant in ploughed fields than in non-inversion or minimum-tillage fields [28,29,30,31], and still positively influenced by conservation practices in semi-natural habitats and rich landscapes [65,82]. In these environments, *Pterostichus* and *Poecilus* are two of the most common genera [31,67], also being abundant in intensively managed agricultural systems and open habitats [64,65,68]. In this study, although *P. melas* was not significantly more abundant, in conservation plots *P. cupreus* was more common, in particular in the plots with vetch as cover crop. Although *P. cupreus* seems to be less efficient in slug control than *Pterostichus* spp. [48,49], it is a polyphagous species able to reduce insect pest populations [51] and associated with slugs in organic farms with green manure [83]. Other Carabidae, both generalist feeders and granivorous (*Bembidion*, *Brachinus*, and *Harpalus*), were more common in conservation plots with vetch as cover crop and, to a lesser extent, with the mixture cover, than in conventionally managed plots. The presence of slugs and ground beetles in traps and refuges can also be influenced by the physical features of the environment in the area of the trap / refuge. For example, soil compaction or the presence of plant residues may influence the locomotion and activity of the target organism, impacting the effectiveness of the monitoring method [84,85].

## 5. Conclusions

The present study focuses on the evaluation of slug and ground beetle monitoring strategies in conservation agriculture and the impact of such strategies on carabid populations. Conservation agriculture can be seriously affected by the presence of pestiferous slugs, and traps are monitoring tools that can be used when planning the timing of molluscicide applications. There was wide variation in the efficacy of the tested slug traps and shelters. In general, wooden boards did provide some information, but pitfall traps using beer as the lure provided far more specimens. However, the use of beer-baited pitfall traps necessitates consideration of the effort required for their maintenance, the risk of disturbance by wild fauna and, above all, their potential impact on populations of beneficial insects such as predatory ground beetles, in particular when the traps are used in large numbers.

*D. invadens*, the most common pestiferous slug found in this study, was more abundant in conventional plots than in conservation plots, and this could be related to the lower presence of natural enemies such as carabids. It should be remembered that these results were obtained in a no-till soil after an appropriate transition period from a previous conventional management and therefore soil biodiversity had already reached a new equilibrium. Indeed, the presence of a considerable community of ground beetles was positively related to conservation practices and, of these, a cover crop of hairy vetch showed the highest positive effect compared to the other treatments, at least for *P. cupreus*. Taken together, these results show that field management might influence the community of natural enemies and the related slug population. The latter’s presence can be monitored by using beer-baited pitfall traps, at least during the most susceptible stages of the crop and in periods of slug activity.

## Figures and Tables

**Table 1:** Mean catches (±SE; *n* = 156) of *D. invadens* and ground beetles (*P. cupreus*, *P. melas* and other Carabidae) in trial no. 1 according to different soil management systems.

Soil Management	*D. invadens*	*P. cupreus*	*P. melas*	Carabidae (Other Genera)
CONV-Plough ^1^	4.9 (±1.1) b	11.7 (±2.2) a	2.3 (±0.4) a	4.7 (±0.9) a
CONS-Rye ^2^	3.3 (±0.8) ab	20.4 (±2.7) b	2.2 (±0.4) a	5.6 (±1.1) a
CONS-Vetch ^3^	1.2 (±0.2) a	27.0 (±4.1) c	2.1 (±0.4) a	9.1 (±1.5) b
CONS-Mix ^4^	1.2 (±0.2) a	18.9 (±2.8) b	3.5 (±0.7) a	7.4 (±1.4) ab

^1^ CONV-Plough = conventional tillage; ^2^ CONS-Rye = no-tillage with cover crop of rye; ^3^ CONS-Vetch = no-tillage with cover crop of hairy vetch; ^4^ CONS-Mix = no-tillage with cover crop of mixture. Means followed by the same letter do not differ at *p* = 0.05 level (Student-Newman-Keuls *post hoc* test).

**Table 2:** Mean catches (±SE, *n* = 208) of *D. invadens* and ground beetles (*P. cupreus*, *P. melas* and other Carabidae) in trial no. 1 according to the different type of trap.

Trap Type	*D. invadens*	*P. cupreus*	*P. melas*	Carabidae (Other Genera)
wooden boards	0.6 (±0.1) a	0.1 (±0.0) a	0.0 (±0.0) a	0.0 (±0.0) a
beer A	3.4 (±0.6) b	27.1 (±3.0) b	3.3 (±0.4) b	9.1 (±1.2) b
beer B	4.0 (±0.8) b	31.3 (±3.1) b	4.3 (±0.6) c	10.9 (±1.3) b

Means followed by the same letter do not differ at *p* = 0.05 level (Student-Newman-Keuls *post hoc* test).

**Table 3:** Mean catches (±SE, *n* = 64) of *D. invadens* and ground beetles (*P. cupreus*, *P. melas* and other Carabidae) in trial no. 2 according to the different type of trap.

Trap Type	*D. invadens*	*P. cupreus*	*P. melas*	Carabidae (Other Genera)
wooden board	0.6 (±0.1) a	0.0 (±1.1) a	0.00 (±0.0) a	0.0 (±0.0) a
beer A	1.5 (±0.6) a	29.1 (±8.0) b	0.16 (±0.1) ab	20.2 (±5.3) b
beer B	1.6 (±0.6) a	36.7 (±10.0) b	0.22 (±0.1) b	24.1 (±6.8) b

Means followed by the same letter do not differ at *p* = 0.05 level (Student-Newman-Keuls *post hoc* tests).

## Supplementary Materials

The following are available online at https://www.mdpi.com/2075-4450/11/6/380/s1, Figure S1: Temperatures and rain events recorded during the experiment period by the nearest meteorological station, Table S1: Trial 1 statistics, Table S2: Trial 2 statistics.
