# Supplementary figures and images for: Slug Monitoring and Impacts on the Ground Beetle Community in the Frame of Sustainable Pest Control in Conventional and Conservation Agroecosystems
Source: Insects. 2020 Jun 18;11(6):380. doi: 10.3390/insects11060380 (PMC7349495; doi:10.3390/insects11060380)

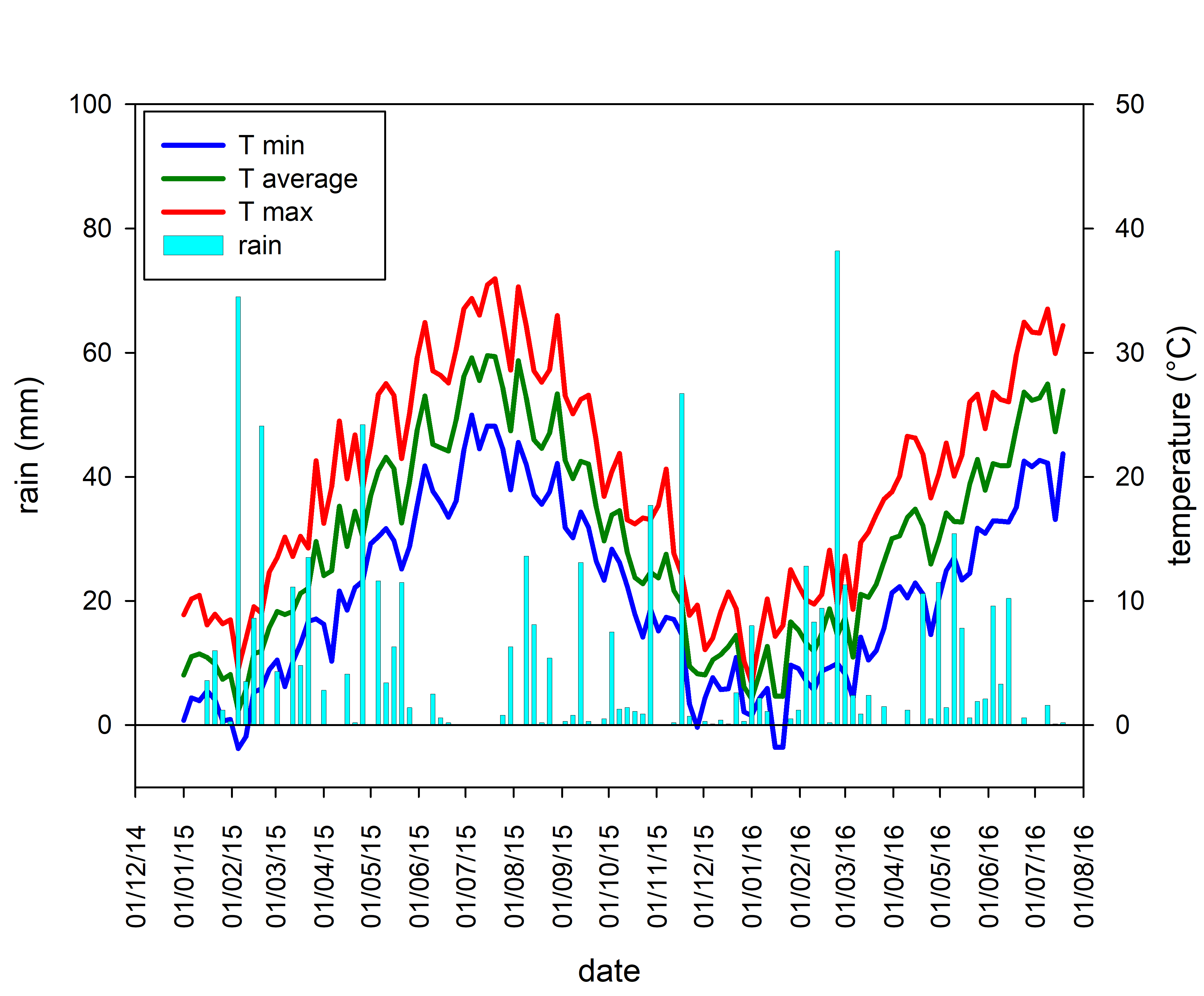

Supplement: Supplementary file 1 [file insects-11-00380-s001.zip › Supplementary Material/Figure S1.TIF]
